# Prediction of insulin treatment in women with gestational diabetes mellitus

**DOI:** 10.1038/s41387-021-00173-0

**Published:** 2021-10-02

**Authors:** Makarios Eleftheriades, Christos Chatzakis, Eftychia Papachatzopoulou, Vassilis Papadopoulos, Irene Lambrinoudaki, Konstantinos Dinas, George Chrousos, Alexandros Sotiriadis

**Affiliations:** 1 2nd Department of Obstetrics and Gynecology Aretaieio Hospital, National and Kapodistrian University of Athens - Faculty of Medicine, Athens, Greece; 2 2nd Department of Obstetrics and Gynecology, Aristotle University of Thessaloniki School of Medicine, Thessaloniki, Greece; 3 University of Patras Medical School, Department of Obstetrics and Gynecology, Patras, Greece; 4 University Research Institute of Maternal and Child Health and Precision Medicine, National and Kapodistrian University of Athens, Medical School, Athens, Greece

**Keywords:** Gestational diabetes, Risk factors

## Abstract

**Introduction:**

The identification of pregnant women with Gestational Diabetes Mellitus (GDM) who will require insulin therapy, may modify their management to closer monitoring and probable early interventions. The aim of the study was to develop a predictive model for the necessity of insulin treatment in women with GDM.

**Materials and methods:**

This was a prospective cohort study. Data from 775 women diagnosed with GDM per the IADPSG criteria were analyzed using logistic regression and a machine learning algorithm, the Classification and Regression Trees (CART). Potential predictors routinely recorded at follow-up visits were tested and used for the development of the model. The resultant model was externally validated using the data from two different perinatology clinics.

**Results:**

Preconceptional maternal BMI and morning fasting blood glucose levels at baseline and at 1 h during an Oral Glucose Tolerance Test (OGTT) were independent significant predictors for the treatment modality of GDM. Baseline blood glucose greater than 98 mg/dl and preconceptional maternal Body Mass Index (BMI) between 26 and 31 kg/height^2^ increased substantially the probability of insulin therapy (odds ratio [OR] 4.04, 95% confidence interval [CI] CI 2.65–6.17 and 2.21, 95%CI 1.42–3.43, respectively). The area under the curve (AUC) for the internal and external validation of the predictive model was 0.74 and 0.77, respectively.

**Conclusions:**

A simple model based on maternal characteristics and the values of an OGTT can predict the need for insulin treatment with accuracy. Overweight women with an abnormal baseline blood glucose at OGTT are at high likelihood for insulin treatment.

**Key message:**

Fifteen to 30% of women with Gestational Diabetes Mellitus (GDM) require insulin therapy. Overweight women with baseline blood glucose greater than 98 mg/dl at OGTT are at increased risk for insulin treatment and close monitoring and increased physical exercise are required.

## Introduction

Glucose intolerance at the onset of or at first recognition during pregnancy is historically defined as Gestational Diabetes Mellitus (GDM) [1]. Some organizations use the term overt diabetes for women with probable preexisting diabetes that is first recognized in early pregnancy and “gestational diabetes” for women with glucose intolerance in the late second or the third trimester respectively. Diagnosis of diabetes at 24–28 weeks of gestation is consistent with “gestational diabetes”, while diagnosis at the first prenatal visit (in early pregnancy) is more consistent with “overt diabetes”. GDM develops in women whose pancreatic function is insufficient to overcome the insulin resistance associated with the pregnant state [2]. GDM is associated with various complications, with the more prominent being an increased risk of spontaneous abortion, fetal anomalies, preeclampsia, fetal macrosomia, cesarean delivery, and neonatal hypoglycemia, along with their associated morbidities [3–7]. Appropriate monitoring and treatment of gestational diabetes can improve pregnancy outcome [8]. Many women can achieve euglycemia and improved pregnancy outcomes with nutritional therapy alone, with only 15% to 30% of women with GDM requiring insulin therapy [9]. However, the American College of Obstetricians and Gynecologists has suggested intensified antenatal maternal and fetal assessments in all women treated with insulin [10]. In addition, employing the new criteria for the diagnosis of gestational diabetes mellitus introduced by the International Association of Diabetes and Pregnancy Study Groups (IADPSG), increased the global prevalence of GDM to about 18 percent [11], which, in turn, increases the need for more efficient and effective monitoring and treatment strategies.

Previous studies have identified clinical and biochemical factors that are associated with the need for insulin therapy in women with GDM at the time of diagnosis [12–20], while a limited number of studies developed predictive models for the necessity of insulin therapy [14, 15, 20, 21]. However, none of these studies used the IADPSG criteria for the diagnosis of GDM.

The aim of this study was to identify clinical, biochemical, and ultrasonographic factors that are associated with the need for insulin therapy in women diagnosed with GDM using the IADPSG criteria and to develop a predictive model.

## Results

Seven hundred and seventy-five women fulfilled the eligibility criteria and were included in the analysis. Of those, 645 (83.2%) were treated with physical exercise and dietary modification and 130 (16.8%) were treated with insulin.

### Descriptive data

**Fig. 1:**
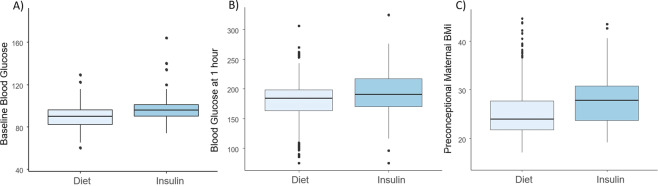
Descriptive statistics for the two groups. Box-and-whiskers plots of (**A**) blood glucose levels at baseline (**B**) blood glucose at 1 h during a 75 gr Oral Glucose Tolerance Test (OGTT), and (**C**) preconceptional maternal BMI in the Diet and Insulin groups.

The descriptive statistics for the two groups are shown in Table 1 and Fig. 1. In terms of maternal characteristics, women in dietary modification and physical exercise had similar rates of smoking and methods of conception with women in the insulin group. Maternal age did not differ between the two groups. However, there was a statistically significant difference between the two groups in the maternal Body Mass Index (BMI) before conception and in the levels of blood glucose during the 75-gram 2-h OGTT at all three-time points of the test. Women that were treated with insulin had greater BMI and higher blood glucose levels during the test (BMI, BG at Baseline and at 1-h *P* < 0.001, BG at 2 h *p* = 0.023).

**Table 1:** Maternal characteristics in Diet and Insulin group.

	DIET	INSULIN	*P*
**Smoking,*n*(%)**	69 (11)	22 (16.5)	0.074
**ART Conception,*n*(%)**	75 (11.9)	17 (12.8)	0.966
**Maternal age, mean (SD)**	34.15 (4.2)	34.54 (4.0)	0.105
**BMI, mean (SD)**	25.22 (5.14)	27.94 (5.44)	<0.001
**T0 (Fasting blood glucose), mean (SD)**	89.2 (9.7)	97.8 (13.3)	<0.001
**T60 (blood glucose at 1** **h), mean (SD)**	179.2 (31.9)	192.9 (36.9)	<0.001
**T120 (blood glucose at 2** **h), mean (SD)**	148.2 (35.1)	158.5 (39.9)	0.023

In order to assess differences between the two groups, *t* test was used for the continuous variables and Chi-square test was used for the qualitative variables.

### Main results

A regression model was used to test for significant covariates in the prediction of treatment for the GDM. The potential predictors included maternal age, smoking, method of conception, maternal BMI before conception, blood glucose levels at baseline, at 1 h and at 2 h during the OGTT, and *z* scores for the fetal abdominal circumference in the second trimester. Maternal BMI and blood glucose values were treated as a continuous variable and there were not divided into categories. Maternal BMI (OR 1.043; 95% CI 1.004–1.082), blood glucose levels at baseline (OR 1.061; 95% CI 1.038–1.084) and at 1 h during the OGTT (OR 1.011; 95% CI 1.018–1.082), were independent significant predictors for the treatment of GDM (Nagelkerke *R*^2^ = 0.172; AUC 0.743, 95%CI 0.7–0.79).

**Fig. 2:**
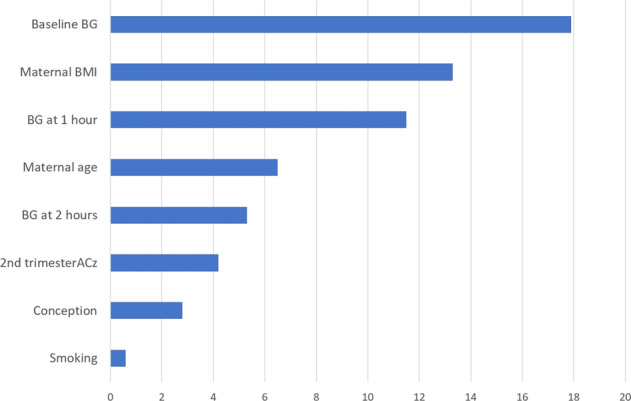
Variable importance. Bar chart of variable importance for the different factors tested in the development of the Classification Tree, using the “variable.importance” command in rpart package.

**Fig. 3:**
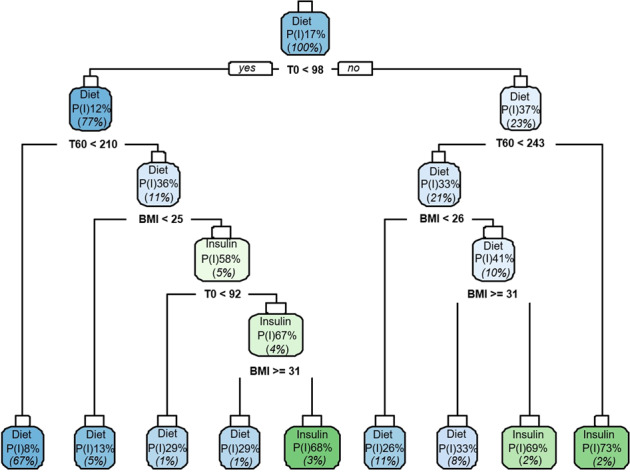
Classification Tree for the prediction of insulin treatment in women with Gestational Diabetes Mellitus (GDM). Blue boxes represent greater likelihood for successful control with dietary and exercise modification, whereas green boxes represent greater likelihood for recourse to insulin treatment. T0 and T60 represent the baseline and the one-hour blood glucose values respectively. For each box, P(I) represents the probability of need for insulin treatment is displayed; the percentage numbers in parentheses (italics) represent the proportion of women in that step.

The same potential factors were entered in a prediction model using a CART algorithm and their importance were displayed (Fig. 2). The final classification tree is shown in Fig. 3. The initial probability of a woman with GDM to be treated with insulin is 16.8%. If the patient’s baseline blood glucose is greater than 98 mg/dl, that probability doubles and reaches the value of 37%. If this finding is in conjunction with 1-h blood glucose greater than 243 mg/dl, the probability reaches the value of 73%. If the 1-hour blood glucose is less than 243 mg/dl and maternal BMI is between 26 and 31, the probability of a woman to be treated with insulin reaches 69%.

Conversely, if maternal baseline blood glucose is less than 98 mg/dl, the probability for insulin treatment drops to 12%. However, if the patient has 1-h blood glucose greater than 210 mg/dl, baseline blood glucose between 85 and 92 mg/dl and BMI between 25 and 31, the probability for insulin treatment increases to 82%.

Overall, women with baseline blood glucose greater than 98 mg/dl and BMI between 26 and 31 were in greater risk for insulin treatment (OR 4.04, 95% CI 2.65–6.17 and OR 2.21, 95%CI 1.42–3.43, respectively).

The internal validation of the predictive model resulted in AUC 0.75 (95%CI 0.70–0.78). The external validation set comprised 168 women who fulfilled the eligibility criteria. Of these, 142 (84.5%) were treated with nutritional therapy and physical exercise and 26 (15.5%) were treated with insulin. The women had a mean age of 34.3 ± 4.3 years, mean blood glucose at baseline 91 ± 11 mg/dl, mean blood glucose at 1 h 181 ± 34 mg/dl, and a preconceptual BMI of 25.8 ± 4.4. External validation of the model resulted in AUC 0.77 95%CI 0.73–0.81).

## Discussion

We developed a predictive model for the necessity of insulin treatment in women with GDM, using CART, a machine learning algorithm. Among the clinical, biochemical, and sonographic factors that were collected, maternal BMI before conception and blood glucose levels at baseline and at 1 h during the OGTT were significant predictive factors. Our model shows that the probability of a pregnant woman with GDM to receive therapy with insulin is substantially increased when patient’s baseline blood glucose is greater than 98 mg/dl and pre-conceptional maternal BMI ranges between 25 and 31.

This study showed that preconceptional maternal BMI and the blood glucose levels at baseline and at 1 h during the OGTT are predictive factors for insulin treatment in women with GDM. Previous studies have also explored potential predictors of insulin treatment [12–20], mostly using multivariate regression analysis [13, 15, 17–20]. As in our study, pre-conceptional maternal BMI [12, 14, 15, 19], and blood glucose levels at baseline [12, 14, 16, 19], and at 1 h during the OGTT [12, 17] have been commonly identified as predictors.

Increased baseline blood glucose could indicate impaired pulsatile and/or continuous insulin production [22, 23]. In pregnancy, compensatory increases in β-cell mass are achieved through a combination of hypertrophic expansion, proliferation, and potentially, neogenesis from precursor cells accompanied by a temporary decrease in apoptosis [24]. Impairment of one or more pathways, can lead to hyperglycemia and GDM [25]. In patients with Impaired Fasting Glucose (IFG) absolute and relative insulin secretion are impaired, possibly due to an underlying β-cell failure or reduced beta-cell mass [26, 27]. In addition, in the presence of peripheral insulin resistance, beta-cell function or mass may progressively decline [26].

We also found that overweight rather than obese women had an increased probability for insulin treatment. Although this finding may be seem counterintuitive, it might be explained by the effect of BMI on insulin secretion. Indeed, there is evidence that in type 2 diabetic patients, BMI has a linear relationship to insulin secretion [28]. Therefore, obese patients have elevated levels of insulin in both the basal state and in response to glucose [28–30]. This is in agreement with a large cohort study which showed that obese individuals may develop diabetes type 2 predominantly through insulin resistance rather through impaired insulin secretion [31].

The observed association between increased baseline blood glucose at OGTT and need for insulin treatment could be attributed to impaired insulin production in these women [22, 23]. Women with GDM identified as high risk for insulin treatment may benefit from physical exercise. The possibility of an underlying pancreatic impairment in women with increased baseline blood glucose at OGTT may decrease the effectiveness of a dietary modification. In contrast, exercise training which increases pancreatic β-cell function in a linear dose-response manner, is recommended in women with poor insulin secretion capacity [32].

This is the only study using the IADPSG criteria [33] to develop a predictive model for the need for insulin treatment in women diagnosed with GDM. The largest previous study used GDM criteria in place since 1991 [14]. Furthermore, three other studies used either the Australian Diabetes in Pregnancy Society criteria [34] or the number of abnormal 100 g glucose 3 h OGTT values at diagnosis [15, 21].

Another strength of this study is its external validation. The data used for the latter were derived from two different prenatal clinics in Greece, which are located in different Greek cities, improving the generalizability and the applicability of our model. External validation has only been performed in one of the previous studies [14] with lower point estimates for accuracy than the present model (AUC 0.71 and 0.707 for the internal and external validation, respectively).

Moreover, this is the only study using a machine learning method, classification, and regression tree (CART), for the prediction of insulin therapy need in women with GDM. The advantage of this method is that our results can guide the clinical decision of the attending physician, providing a structured and easy to interpret algorithm. In every step of the resultant algorithm results, the probability of a woman’s need to be treated with insulin is displayed.

Finally, this predictive model is easy to implement and interpret, as it is based on data routinely collected at follow-up visits of pregnant women and results in a comprehensive probability tree.

The main limitation of the study is that it was an interventional trial, the decision for insulin treatment was at the clinician’s discretion and was based on concurrent clinical guidelines rather than a predefined study protocol. On the other hand, the pragmatic conditions of clinical decision making enhance the clinical generalizability of the model, which was confirmed by the results of the external validation.

A simple model based on maternal characteristics and the glucose values of an OGTT can predict the need for insulin treatment with fair accuracy. Overweight women with abnormal baseline or stimulated blood glucose at OGTT appear to be at high risk for insulin treatment. Close monitoring and increased physical exercise might potentially be an option for them.

## Methods

### Study design

We followed prospectively a cohort of pregnant women with singleton pregnancy and gestational diabetes mellitus, who attended the prenatal clinic, and in whom we had the pregnancy outcome.

### Setting

This study carried out in the prenatal clinic of Aretaeion Hospital of the National and Kapodistrian University of Athens between 2010 and 2018. The data of women with GDM were prospectively collected as part of routine heath care and then entered in a fetal database (Astraia software, Munich, Germany). These included demographic, clinical, laboratory, and ultrasonographic data of pregnant women and their fetuses.

### Participants

Women with singleton pregnancies, who were diagnosed with gestational diabetes mellitus were included in the study. Diagnosis of diabetes was established according to International Association of Diabetes and Pregnancy Study Groups (IADPSG) criteria [33]. More specifically, women between 24 and 28 weeks of gestation had a 75-g 2-h oral glucose tolerance test. If their blood glucose (BG) values during the test were equal or greater than 92 mg/dl at fasting, 180 mg/dl 1 h after the glucose ingestion, or 153 mg/dl 2 h after the glucose ingestion, the diagnosis of GDM was established. Women with preexisting diabetes mellitus or twin pregnancies were excluded from the study.

All women gave their informed consent that their anonymized data could be used for research purposes. The study was reviewed and approved by the institutional review board of the Aretaieio Hospital, National & Kapodistrian University of Athens, Greece. All methods were performed in accordance with the Declaration of Helsinki.

### Variables

After the diagnosis, women received nutritional and physical exercise counseling as the first-line therapy. Insulin was commenced if optimal glycemic targets (defined as fasting blood glucose <95 mg/md, 1 h postprandial blood glucose <140 mg/dl, and 2 h postprandial blood glucose <120 mg/dl) were not reached within two weeks (more than 30 percent of the recorded values out of the optimal range). Moreover, insulin was started at any time during pregnancy when a sustained achievement of glycemic targets by nutritional therapy and physical exercise failed to occur. Oral hypoglycemic agents were not used. Therefore, two main groups were formed. Women with GDM in the non-insulin group who achieved optimal glycemia following nutritional therapy and physical exercise, and women in the insulin therapy group who were treated with insulin to achieve an optimal glycemic control.

All ultrasound examinations were performed by Fetal Medicine Foundation (www.fetalmedicine.com) certified sonographers and dating of the pregnancy was based on CRL. Maternal serum free β-human chorionic gonadotrophin (free β-hCG) and maternal serum pregnancy-associated plasma protein A (PAPP-A) were recorded and transformed into multiple of the median (MoM). In addition, sonographic measurements, including the estimated fetal weight and the abdominal circumference were recorded and transformed into percentiles. Maternal and pregnancy characteristics also were recorded, including ethnicity, maternal age, maternal height, weight and BMI, medical history, smoking status, alcohol consumption, parity, method of conception. All the data have been recorded in the Astraia software (Astraia GmbH, Munich, Germany).

### Data sources/measurement

Data on the outcome of GDM treatment method were collected through communication with the mothers during their follow-up visits. Clinical, biochemical, and sonographic parameters were retrieved from the department’s archives.

### Statistical methods

Continuous variables were presented as mean ± SD, if their distribution was normal, or as medians and interquartile range values if the distribution was non-normal. Categorical variables were summarized as percentages, together with their 95% confidence intervals (95% CIs). The Student t-test or the Mann-Whitney test were used for comparisons between the two groups. Chi-square or Fisher’s exact test was used for pairwise comparisons of proportions, as appropriate, and odds ratios (ORs) along with their 95% CIs were calculated. If the assumptions of both the chi-square and the Fisher’s exact test were violated, only ORs were used. In all the above tests, a *p* value of <0.05 was considered significant.

Logistic regression (backward, by likelihood ratios) was performed for the outcome of Insulin/dietary modification and physical exercise. Potential predictors included maternal characteristics, laboratory results, and clinical and sonographic findings.

### Classification and regression tree (CART)

Furthermore, a machine-learning algorithm the Classification and Regression Tree (CART) was employed to develop a predictive model for the treatment that a woman with GDM would need to follow. The same potential predictive factors used in the development of logistic regression were included in the model and the classification tree was developed. CARTs have several advantages as predictive models [35–37]. First, the resultant algorithms are easy to interpret, as they represent a sequential method that results in the determination of the optimal algorithm. Second, in every step of the algorithm, the probability of a case belonging to a specific group is known. Thus, the clinician can decide on the therapy course if the results are satisfactory. To train the data and grow a tree, split functions are identified that evaluate features from the training dataset and pass to a left or right branch of the tree. The split functions are calculated to produce the best split separating the class labels. The data are passed down the tree and the tree grows from the root (starting point) to a terminal branch, which is called a leaf [35]. For the model produced by the decision tree, predicted probabilities are used for the assessment of the accuracy, expressed by Receiver Operating Characteristics (ROC) curves and areas under the curve (AUC).

The model was internally and externally validated. For the internal validation of the model, data from the original dataset were randomly sampled using 10 folds cross validation. For the external validation of the model, data of women with GDM who attended the prenatal clinic of two different Departments (Second Department of Obstetrics and Gynecology, Aristotle University of Thessaloniki, and Department of Obstetrics and Gynecology, Patras University) were used. In both clinics, the diagnosis of diabetes was established according to IADPSG criteria, and subsequent management consisted of dietary modification and physical exercise, followed by insulin, if glycemic control was not achieved within two weeks.

The analyses were performed on SPSS (IBM Corp. Released 2016. IBM SPSS Statistics for Windows, Version 24.0. Armonk, NY: IBM Corp.) and open source software R 2.15.1 (The R Foundation for Statistical Computing), using the “rpart” package [38].

## References

[1] Metzger BE, Coustan DR (1998). Summary and recommendations of the Fourth International Workshop-Conference on Gestational Diabetes Mellitus. The Organizing Committee. Diabetes Care.

[2] Proceedings of the 4th International Workshop-Conference on Gestational Diabetes Mellitus. Chicago, Illinois, USA. 14-16 March 1997. Diabetes Care 1998;21:B1-167. 9841138

[3] Yogev H (2010). Hyperglycemia and Adverse Pregnancy Outcome (HAPO) study: preeclampsia. Am J Obstet Gynecol.

[4] Kwik M, Seeho SKM, Smith C, McElduff A, Morris JM (2007). Outcomes of pregnancies affected by impaired glucose tolerance. Diabetes Res Clin Pr.

[5] Bérard J, Dufour P, Vinatier D, Subtil D, Vanderstichèle S, Monnier JC (1998). Fetal macrosomia: risk factors and outcome. A study of the outcome concerning 100 cases >4500 g. Eur J Obstet Gynecol Reprod Biol.

[6] Stotland NE, Caughey AB, Breed EM, Escobar GJ (2004). Risk factors and obstetric complications associated with macrosomia. Int J Gynaecol Obstet.

[7] Yogev Y, Xenakis EMJ, Langer O (2004). The association between preeclampsia and the severity of gestational diabetes: the impact of glycemic control. Am J Obstet Gynecol.

[8] Hartling L, Dryden DM, Guthrie A, Muise M, Vandermeer B, Donovan L (2013). Benefits and harms of treating gestational diabetes mellitus: a systematic review and meta-analysis for the U.S. Preventive Services Task Force and the National Institutes of Health Office of Medical Applications of Research. Ann Intern Med.

[9] American Diabetes Association. (2019). 14. Management of Diabetes in Pregnancy: Standards of Medical Care in Diabetes-2019. Diabetes Care.

[10] Committee on Practice Bulletins—Obstetrics. ACOG Practice Bulletin No. (2018). 190: Gestational Diabetes Mellitus. Obstet Gynecol.

[11] Guariguata L, Linnenkamp U, Beagley J, Whiting DR, Cho NH (2014). Global estimates of the prevalence of hyperglycaemia in pregnancy. Diabetes Res Clin Pr.

[12] McFarland M (1999). Dietary therapy for gestational diabetes: how long is long enough?. Obstet Gynecol.

[13] Zhang Y, Shao J, Li F, Xu X (2016). Factors in Gestational Diabetes Mellitus Predicting the Needs for Insulin Therapy. Int J Endocrinol.

[14] Barnes RA, Wong T, Ross GP, Jalaludin BB, Wong VW, Smart CE (2016). A novel validated model for the prediction of insulin therapy initiation and adverse perinatal outcomes in women with gestational diabetes mellitus. Diabetologia.

[15] Sapienza AD, Francisco RPV, Trindade TC, Zugaib M (2010). Factors predicting the need for insulin therapy in patients with gestational diabetes mellitus. Diabetes Res Clin Pr.

[16] Akinci B, Celtik A, Yener S, Yesil S (2008). Is fasting glucose level during oral glucose tolerance test an indicator of the insulin need in gestational diabetes?. Diabetes Res Clin Pr.

[17] Mitra S, Nayak PK, Sahoo J, Mathew A, Padma A, Kamalanathan S (2014). Predictors for antenatal insulin requirement in gestational diabetes. Gynecol Endocrinol.

[18] Bakiner O, Bozkirli E, Ozsahin K, Sariturk C, Ertorer E (2013). Risk Factors That can Predict Antenatal Insulin Need in Gestational Diabetes. J Clin Med Res.

[19] Wong VW, Jalaludin B (2011). Gestational diabetes mellitus: Who requires insulin therapy?. Aust N. Zeal J Obstet Gynaecol.

[20] Pertot T, Molyneaux L, Tan K, Ross GP, Yue DK, Wong J (2011). Can common clinical parameters be used to identify patients who will need insulin treatment in gestational diabetes mellitus?. Diabetes Care.

[21] Mendez-Figueroa H, Daley J, Lopes V, Coustan D (2013). Predicting the Need for Medical Therapy in Patients with Mild Gestational Diabetes. Am J Perinatol.

[22] Lang DA, Matthews DR, Burnett M, Turner RC (1981). Brief, irregular oscillations of basal plasma insulin and glucose concentrations in diabetic man. Diabetes.

[23] Thompson RS, Rivara F, Thompson D (1989). The New England Journal of Medicine Downloaded from nejm.org at UC SHARED JOURNAL COLLECTION on April 8, 2014. For personal use only. No other uses without permission. From the NEJM Archive. Copyright © 2010 Massachusetts Medical Society. All rights reser. N. Engl J Med.

[24] Butler AE, Cao-Minh L, Galasso R, Rizza RA, Corradin A, Cobelli C (2010). Adaptive changes in pancreatic beta cell fractional area and beta cell turnover in human pregnancy. Diabetologia.

[25] Moyce BL, Dolinsky VW Maternal β-Cell Adaptations in Pregnancy and Placental Signalling: Implications for Gestational Diabetes. *Int J Mol Sci* 2018; **19**. 10.3390/ijms19113467. 10.3390/ijms19113467 PMC6274918 30400566

[26] Færch K, Borch-Johnsen K, Holst JJ, Vaag A (2009). Pathophysiology and aetiology of impaired fasting glycaemia and impaired glucose tolerance: Does it matter for prevention and treatment of type 2 diabetes?. Diabetologia.

[27] Butler AE, Janson J, Bonner-Weir S, Ritzel R, Rizza RA, Butler PC (2003). Beta-cell deficit and increased beta-cell apoptosis in humans with type 2 diabetes. Diabetes.

[28] Bagdade JD, Bierman EL, Porte D (1967). The Significance of Basal Insulin Levels in the Evaluation of the Insulin Response to Glucose in Diabetic and Nondiabetic Subjects. J Clin Invest.

[29] Mohan V, Amutha A, Ranjani H, Unnikrishnan R, Datta M, Anjana RM (2013). Associations of *β* -Cell Function and Insulin Resistance with Youth-Onset Type 2 Diabetes and Prediabetes Among Asian Indians. Diabetes Technol Ther.

[30] Chung JO, Cho DH, Chung DJ, Chung MY (2012). Associations among body mass index, insulin resistance, and pancreatic β-cell function in Korean patients with new-onset type 2 diabetes. Korean J Intern Med.

[31] Tatsumi Y, Morimoto A, Miyamatsu N, Noda M, Ohno Y, Deura K (2015). Effect of Body Mass Index on Insulin Secretion or Sensitivity and Diabetes. Am J Prev Med.

[32] Malin SK, Solomon TP, Blaszczak A, Finnegan S, Filion J, Kirwan JP (2013). Pancreatic β-cell function increases in a linear dose-response manner following exercise training in adults with prediabetes. Am J Physiol Endocrinol Metab.

[33] Metzger BE, Gabbe SG, Persson B, Buchanan TA, Catalano PA, Damm P, International Association of Diabetes and Pregnancy Study Groups Consensus Panel IA of D and PSGC (2010). International association of diabetes and pregnancy study groups recommendations on the diagnosis and classification of hyperglycemia in pregnancy. Diabetes Care.

[34] Hoffman L, Nolan C, Wilson JD, Oats JJ, Simmons D (1998). Gestational diabetes mellitus—management guidelines. The Australasian Diabetes in Pregnancy Society. Med J Aust.

[35] Hastie T, Tibshirani R, Friedman J Springer Series in Statistics The Elements of Statistical Learning Data Mining, Inference, and Prediction. https://web.stanford.edu/~hastie/Papers/ESLII.pdf (accessed 9 Mar2019).

[36] Vach W *Regression Models as a Tool in Medical Research*. Chapman and Hall/CRC, 2012. 10.1201/b12925.

[37] Abellán J, Masegosa AR (2009). An Experimental Study about Simple Decision Trees for Bagging Ensemble on Datasets with Classification Noise.

[38] Therneau TM, Atkinson BRM The rpart package. 2010.

